# A Nomogram Model Based on Neuroendocrine Markers for Predicting the Prognosis of Neuroendocrine Carcinoma of Cervix

**DOI:** 10.3390/jcm12031227

**Published:** 2023-02-03

**Authors:** Mingzhu Jia, Jiangchuan Pi, Juan Zou, Min Feng, Huiling Chen, Changsheng Lin, Shuqi Yang, Ying Deng, Xue Xiao

**Affiliations:** 1 Department of Gynecology and Obstetrics, West China Second University Hospital, Sichuan University, Chengdu 610041, China; 2 Department of Urology, Chengdu Second People’s Hospital, Chengdu 610041, China; 3 Department of Gynecology and Obstetrics, The First Affiliated Hospital of Chongqing Medical University, Chongqing 400016, China; 4 Key Laboratory of Birth Defects and Related Diseases of Women and Children (Sichuan University), Ministry of Education, West China Second Hospital, Sichuan University, Chengdu 610041, China

**Keywords:** neuroendocrine carcinoma of cervix, nomogram model, recurrence, neuroendocrine markers, classical parameters

## Abstract

Background: Combining traditional clinical parameters with neuroendocrine markers to construct a nomogram model to predict the postoperative recurrence of neuroendocrine carcinoma of cervix (NECC). Methods: A total of 257 patients were included in this study. Univariate and multivariate Cox regression analyses were used to establish a nomogram model in the training cohorts, which was further validated in the validation cohorts. The calibration curve was used to conduct the internal and external verification of the model. Results: Overall, 41 relapse cases were observed in the training (23 cases) and validation (18 cases) cohorts. The univariate analysis preliminarily showed that FIGO stage, stromal invasion, nerve invasion, lymph vascular space invasion, lymph node involvement, cervical–uterine junction invasion and CgA were correlated with NECC recurrence. The multivariate analysis further confirmed that FIGO stage (*p* = 0.023), stromal invasion (*p* = 0.002), lymph vascular space invasion (*p* = 0.039) and lymph node involvement (*p* = 0.00) were independent risk factors for NECC recurrence, which were ultimately included in the nomogram model. In addition, superior consistency indices were demonstrated in the training (0.863, 95% CI 0.784–0.942) and validation (0.884, 95% CI 0.758–1.010) cohorts. Conclusions: The established nomogram model combining traditional clinical parameters with neuroendocrine markers can reliably and accurately predict the recurrence risks in NECC patients.

## 1. Introduction

Neuroendocrine carcinoma of the cervix (NECC) is a rare and special type of gynecological malignant tumor, and its incidence accounts for about 1–5% of cervical malignant tumors [1,2,3,4,5]. The National Cancer Institute of America classifies neuroendocrine carcinoma of the cervix into four subtypes: atypical carcinoid, typical carcinoid, small cell neuroendocrine carcinoma and large cell neuroendocrine carcinoma [6]. According to the biological invasion characteristics of neuroendocrine carcinoma of the cervix, the World Health Organization updated the classification in 2014, classifying the atypical carcinoid and typical carcinoid as low-grade neuroendocrine carcinomas, and small-cell neuroendocrine carcinoma and large-cell neuroendocrine carcinoma as high-grade neuroendocrine carcinomas [7,8,9]. Different from other cervical squamous epithelial cancers in the same period, cervical neuroendocrine carcinoma is characterized as highly invasive and malignant, especially high-grade neuroendocrine carcinoma, which is more prone to lymph node and distant metastasis, leading to disease progression and tumor recurrence. The overall survival rate of patients is poor, even in the early stage, with the 5-year survival rate reported at only 4–51% [10,11,12]. Therefore, it is of great clinical significance to improve NECC prognosis by improving the early screening of patients with high risk of relapse and taking early intervention measures.

With NECC being a relatively rare cancer type and with no clear consensus reached on its treatment plan, a comprehensive treatment including radical surgery combined with radiotherapy and chemotherapy is often used for clinical management of the disease [13]. At present, the related research studies on the prognosis of cervical neuroendocrine carcinoma are mostly confined to the simple exploration of the risk factors leading to its poor prognosis. The prognosis is roughly evaluated according to whether the patient is accompanied by a certain high-risk factor, but there is a lack of comprehensive analyses of these risk factors, which leads to a lack of a reliable reference for predicting the recurrence risk of the disease. Therefore, we urgently need a new method to quantitatively evaluate the recurrence risk. In the past, some scholars have used traditional clinical parameters to establish a model to predict the prognosis of cervical neuroendocrine carcinoma. For example, Ru Huang et al. used age, surgery, radiotherapy, chemotherapy, tumor size, lymph node involvement and other indicators to construct a nomogram model to predict the overall survival rate of cervical neuroendocrine cancer [14]. Shi-Wen Zhang et al. established a model to predict the recurrence of cervical neuroendocrine carcinoma based on stromal invasion, nerve invasion, parauterine invasion, SOX and P16 [15].

With the development of molecular biology, immunohistochemical markers have been incorporated into the research of tumor diagnosis, treatment and prognosis. Chromogranin A (CgA), neural cell adhesion molecule (CD56); neuron-specific enolase (NSE); and synaptophysin (Syn) are the most common neuroendocrine markers that can be detected by immunohistochemistry, which are frequently used in the differential diagnosis of neuroendocrine carcinoma [4,9,16,17,18]. In recent years, it has been found that these neuroendocrine markers are significantly correlated with tumor recurrence, and they are viewed as potential prognostic markers [19]. However, it is very rare to use the combination of traditional clinical parameters and neuroendocrine markers to collectively construct a model for predicting the recurrence of cervical neuroendocrine carcinoma after operation.

Therefore, this study combines traditional clinical parameters with neuroendocrine markers to establish a new model for predicting the postoperative recurrence of cervical neuroendocrine carcinoma. In doing so, we aim to quantitatively evaluate the postoperative recurrence risk and formulate individualized treatment plans for patients, thereby reducing the disease progression and recurrence rates of cancer, ultimately improving their overall quality of life.

## 2. Materials and Methods

### 2.1. Research Population

The clinical data of patients diagnosed with cervical neuroendocrine carcinoma from 1 January 2010 to 1 January 2021 at the West China Second Hospital of Sichuan University and First Affiliated Hospital of Chongqing Medical University were collected retrospectively. Considering that the International Federation of Gynecology and Obstetrics (FIGO) updated the old staging system in 2009 to the new staging system in 2018, and included the status of lymph node metastasis in the staging, this study restaged the patients according to the new FIGO staging system. All patients received the standard surgical treatment including total hysterectomy and bilateral salpingo-ovariotomy with or without pelvic lymph node or abdominal aortic lymph node resection. The inclusion criteria applied were as follows: 1. patients without preoperative radiotherapy or chemotherapy; 2. primary cervical neuroendocrine carcinoma diagnosed by pathology after operation. The exclusion criteria were as follows: 1. patients without regular follow-up; 2. patients’ failure to conduct standard surgical methods; 3. non-primary cervical neuroendocrine carcinoma; 4. patients with other malignant tumors or fatal complications; 5. incomplete data. All basic information, clinicopathological data and expression levels of immunohistochemical markers of patients were recorded. This study was approved by the Ethics Committees of West China Second Hospital of Sichuan University and First Affiliated Hospital of Chongqing Medical University. Informed consent was obtained from all patients, which conforms to the ethical standards of the Helsinki Declaration.

### 2.2. Immunohistochemistry

The specimens were fixed in formalin immediately after surgery, and pathological analysis was carried out by the same institution through a unified standard procedure. Firstly, samples were made into the formalin-fixed and paraffin-embedded specimens. Secondly, H&E staining was used to confirm the lesions and a2 mm diameter dot to stand for the whole sample was set. Then, the immunohistochemistry (IHC) of Syn, P16, CgA and CD56 was performed on an automated immunostainer (Leica Bond-Max, Milton Keynes, UK). The monoclonal antibodies bought from Maixin, such as anti-P16INK4A (MX007), anti-Synaptophysin (MX038), anti-Chromogranin A (MX018) and anti-CD56 (MX039), were used in IHC. The results of tumor type, tumor differentiation, lesion size, range of invasion and positive staining percentage of immunohistochemical markers were initially interpreted by a professional junior pathologist, and then reviewed by a supervising physician. Immunohistochemical results were independently evaluated by two experienced pathologists. If the proportion of positive tumor cells was no more than 10%, the evaluation was considered consistent; however, if the proportion difference exceeded 10% or +, reevaluation by a third pathologist would be conducted. The definition of positive staining used a four-point scale and was described as follows: staining was graded as 0; 1+ (less than 5% tumor cells with positive staining); 2+ (5–50% tumor cells with positive staining); and 3+ (more than 50% tumor cells with positive staining), respectively.

### 2.3. Follow-Up and Recurrence

The patients were followed up every 3–6 months for 2 years after operation, every 6–12 months in the next 3 years, and every year after 5 years. The strategy of follow-up included regular gynecological examination, serum tumor markers, cervical cytology, cervical biopsy and diagnostic imaging detection. The recurrence was determined by diagnostic imaging detection and pathological examination. Local recurrence was identified as those located in the vagina or pelvic cavity while distant recurrence included lymph node metastases near the abdominal aorta, abdominal cavity or other organs. Recurrence-free survival (RFS) was defined as the postoperative time from surgery to either the patient’s NECC recurrence or end of follow-up, while overall survival (OS) was defined as the postoperative time from surgery to either the patient’s death or end of follow-up.

### 2.4. Statistical Analysis

All analyses for this retrospective research were mainly conducted using R and SPSS software (IBM SPSS 26). Continuous variables were represented by median or average ± standard deviation, and the differences were compared by *t* test or rank sum test. The categorical variables were expressed as frequencies and percentages, with the chi-square test used for comparing between groups. A two-sided *p* < 0.05 was considered statistically significant.

In the training cohort, univariate and multivariate Cox regression analyses were used to evaluate the independent risk factors correlated with the recurrence of cervical neuroendocrine carcinoma. The factors with *p* < 0.05 were further included to establish the nomogram model by R software. The 1-year, 2-year, 3-year and 5-year recurrence-free survival rates of patients were calculated using the sum of risk factor scores based on the established model. The internal and external model validation was subsequently carried out in the training and validation cohorts, respectively. The c-index and calibration curve were used to validate the accuracy of the model in predicting recurrence. The c-index can quantitatively predict the consistency of occurrence probability between the actual and expected events with estimated values equal to 0.50–0.70, 0.71–0.90 and more than 0.90, considered as low, moderate and high accuracy, respectively. Finally, the ROC curve was used to calculate the 3-year recurrence-free survival rate of patients to determine the optimal threshold of this nomogram model. Applying this threshold, the patients in the training group were divided into the High-RFS and Low-RFS groups, with the difference of recurrence-free survival between the two groups compared using the Kaplan–Meier curve.

## 3. Results

### 3.1. Clinicopathological Characteristics of Patints

In this study, the clinical records of 269 hospitalized patients with cervical neuroendocrine carcinoma who underwent surgery were collected. Based on the evaluation of the inclusion and exclusion criteria, 257 patients were identified as the final subjects in the study, including 171 patients from West China Second Hospital of Sichuan University assigned as the training cohort, and 86 patients from the First Affiliated Hospital of Chongqing Medical University assigned as the validation cohort. The average age of disease onset was 45.92 and 44.91 years in the training and validation groups, respectively. In the training cohort, there were 111 cases (64.9%) under stage I, 24 cases (14%) under stage II and 36 cases (21.1%) under stage III, while in the validation cohort, there were 51 cases under stage I (59.3%), 16 cases under stage II (18.6%) and 19 cases under stage III (22.1%). In the training cohort, there were 90 cases (52.6%) reported HPV-positive, while in the validation cohort, 49 cases (57.0%) were reported HPV-positive. For the entire follow-up period, 23 patients (13.5%) relapsed, and 21 patients (12.3%) died in the training cohort, while 18 patients (20.9%) relapsed, and 9 patients (10.5%) died in the verification group. The median time of recurrence-free survival and follow-up of the training group were 42 (2–150) and 44 (2–150) months, respectively, while those for the validation group were 37.5 (4–145) and 38 (4–145) months, respectively. Complete details are provided in Table 1.

#### 3.1.1. Factors Related to Recurrence of Cervical Neuroendocrine Carcinoma

In this study, the univariate Cox regression analysis was used to screen the traditional clinical parameters and neuroendocrine markers (Syn, CgA, CD56) that may potentially affect the recurrence of cervical neuroendocrine carcinoma. Factors with *p* > 0.05 were not included in the multivariate analysis, including age (*p* = 0.401), BMI (*p* = 0.647), endometrial invasion (*p* = 0.066), pathological type (*p* = 0.555), nerve invasion (*p* = 0.269) and Syn (*p* = 0.877). However, factors with *p* < 0.05 including stage, stromal invasion, nerve invasion, lymph vascular space invasion, lymph node metastasis, cervical–uterine junction invasion and CgA were further analyzed using multivariate regression analysis. Results showed that stage, stromal invasion, lymph vascular space invasion, lymph node metastasis, cervical–uterine junction invasion and CgA were confirmed as independent risk factors of cancer recurrence (Table 2).

#### 3.1.2. Predictive Nomogram Model for Cancer Recurrence

**Figure 1:**
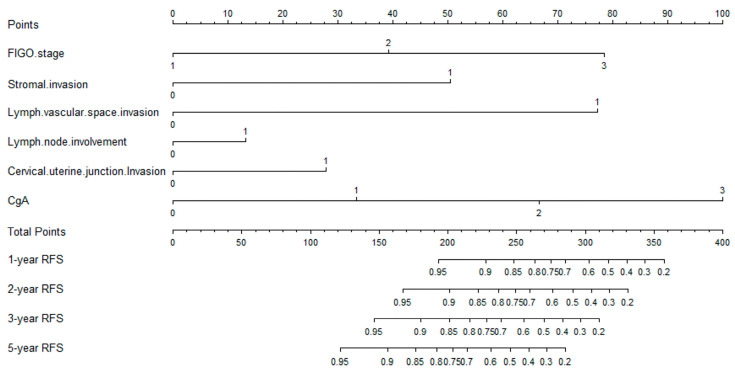
The nomogram model for predicting the recurrence-free survival of neuroendocrinal carcinoma of cervix after surgery. (Each variable from the second row to the seventh row corresponds to a point value in the first row “point”. Then, sum the scores of each variable to obtain the “total point”, and finally derive 1-year, 2-year, 3-year and 5-year recurrence-free survival (RFS)).

**Figure 2:**
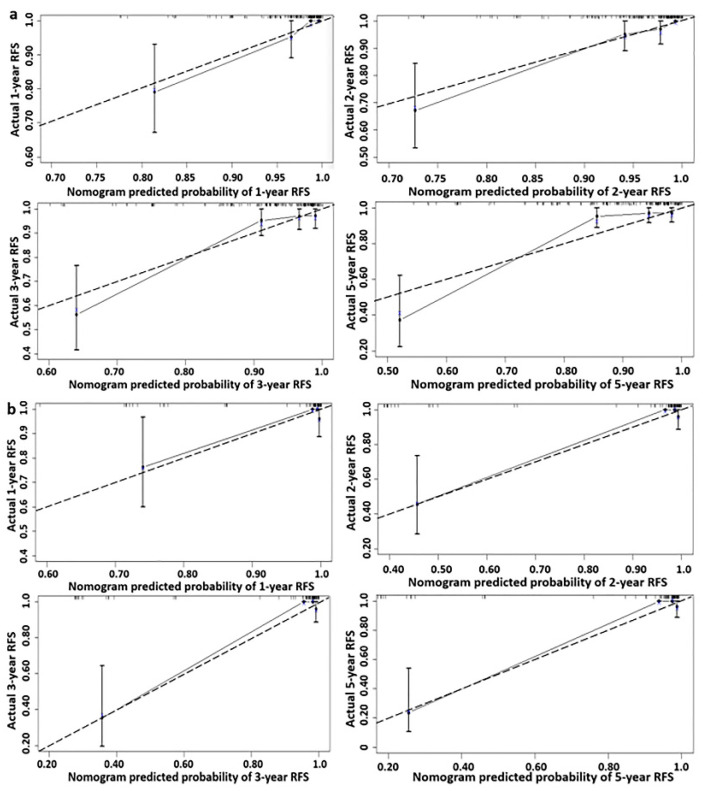
(**a**) The calibration curves of this nomogram model for predicting recurrence-free survival of neuroendocrinal carcinoma of cervix in the training cohort. (**b**) The calibration curves of this nomogram model for predicting recurrence-free survival of neuroendocrinal carcinoma of cervix in the validated cohort. (The dotted line: reference line. The solid line: the prediction curve provided by the nomogram model).

The nomogram model for predicting the recurrence of cervical neuroendocrine carcinoma is shown in Figure 1, which estimates the recurrence-free survival rate (RFS) of patients in a more accurate and simple manner. Each risk factor from the final multivariate regression model was listed separately, with a corresponding number of points assigned to the given magnitude of the risk factor. Then, the cumulative point score for all the risk factors were matched to a scale of outcome, here represented as the patient’s 1-, 2-, 3- and 5-year RFS. In addition, the calibration curves shown in Figure 2 demonstrated excellent model goodness-of-fit performance in both the training and validation groups. Compared with models only employing traditional clinical parameters, the C-indices for this model that included neuroendocrine markers were superior in both cohorts, noted as 0.863 (95% CI, 0.784–0.942) and 0.884 (95% CI, 0.758–1.010) in the training and validation groups, respectively (Table 3).

#### 3.1.3. Optimal Threshold of Recurrence-Free Survival Rate of Nomogram Model

**Figure 3:**
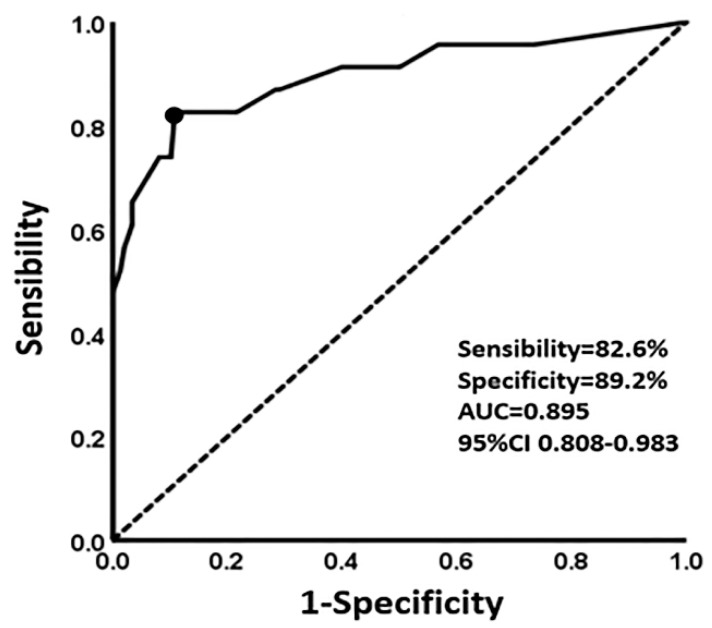
The ROC curve of the optimal threshold of 3–year recurrence-free survival based on the established nomogram model. (Black dot: the area under the curve (AUC) at this point is the largest, which indicates the optimal threshold value of the 3-year recurrence-free survival predicted by the model is 0.85 (sensitivity, 82.6%; specificity, 89.2%; area under the curve = 0.895; 95% CI 0.808–0.983). Dotted line: reference line. Solid line: the ROC curve of the established nomogram model).

**Figure 4:**
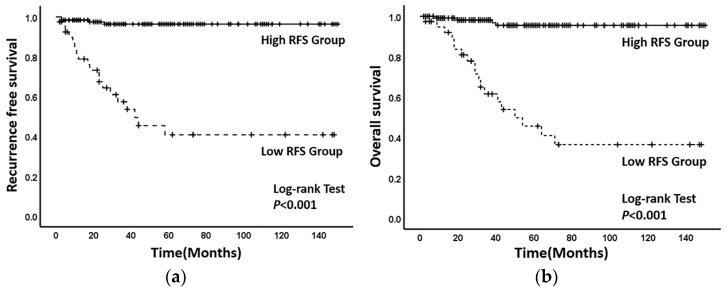
(**a**) Kaplan–Meier survival curves of recurrence-free survival between the low-RFS group and the high-RFS group. (**b**) Kaplan–Meier survival curves of overall survival between the low-RFS group and the high-RFS group. ((**a**) Solid line: the recurrence-free survival curve of the high-RFS group. Dotted line: the recurrence-free survival of the curve of the low-RFS group. (**b**) The solid line: The overall survival curve of the high-RFS group. The dotted line: The overall survival of curve the low-RFS group).

The 3-year recurrence-free survival (RFS) for each patient could be calculated through this established nomogram model, with the optimal threshold determined as 84.5% from the ROC curve (sensitivity = 82.6%, specificity = 89.2%, area under the curve = 0.895 95% CI (0.808–0.983) (Figure 3). Applying the optimal threshold, patients were divided into two groups—patients with RFS < 85% were classified under the low-RFS group, while patients with RFS ≥ 85% were classified under the high-RFS group. The median follow-up time for the low-RFS group was 29 (2–148) months and the recurrence-free survival time was 34 (2–148) months, while those under the high-RFS group all reported 48 (2–150) months. The 3-year recurrence-free survival rates of the patients under the high-RFS and low-RFS groups were 96.6% (95% CI, 94.9–98.3%) and 54.3% (95% CI, 45.5–63.1%), respectively (*p* < 0.001) (Figure 4a). The 3-year overall survival rates for the high-RFS and low-RFS groups were 97.4% (95% CI, 95.5–98.9%) and 64.1% (95% CI, 55.7–72.5%), respectively (*p* < 0.001) (Figure 4b).

## 4. Discussion

Neuroendocrine carcinoma of the cervix is a very rare type of gynecological tumor. Considering its extreme malignancy, rapid disease progression and the lack of reliable prospective research, disease management and treatment remain as clinical challenges. Therefore, it is crucial to improve the prognosis of patients with cervical neuroendocrine carcinoma by strengthening the cognition of the risk factors leading to disease recurrence and optimizing its treatment strategy. Neuroendocrine markers are commonly used to assist in the differential diagnosis of cervical neuroendocrine carcinoma. In recent years, its value in predicting the prognosis of cervical neuroendocrine carcinoma continues to remain significant. However, at present, the models used to predict the prognosis of cervical neuroendocrine carcinoma involving neuroendocrine markers are very rare, so we urgently need a new model to reliably and accurately predict cancer recurrence.

In this study, the univariate and multivariate Cox regression analyses showed that patients with advanced stage, stromal invasion, cervical uterine junction invasion, lymph vascular space invasion and lymph node metastasis had higher risks of recurrence with worst prognoses. In addition, based on simple, fast and low-cost immunohistochemical technology, immunohistochemical markers remain an important part of postoperative histopathological examination. In effect, three commonly used neuroendocrine markers (Syn, CgA, CD56), which can be detected through immunohistochemistry were included in this study. Results showed that patients with CgA positive had generally poor prognoses and were therefore included in this prediction model. Based on the nomogram model we developed, we can roughly predict the postoperative recurrence of each patient with cervical neuroendocrine carcinoma. As an illustration, a patient diagnosed with stage II cancer (39 points), with cervical stromal invasion (50 points), cervical uterine junction invasion (28 points), lymph vascular space invasion (77 points), positive lymph node metastasis (13 points) and CgA 2+ (32 points) will have corresponding 1-, 2-, 3- and 5-year recurrence-free survival rates of 87%, 79%, 69% and 52%, respectively (the probability of recurrence in 1, 2, 3 and 5 years was 13%, 21%, 31% and 48%). Our model could objectively quantify postoperative recurrence instead of simply generalizing recurrence rate as “high or low” by several factors. Having been able to reliably calculate the 1-, 2-, 3- and 5-year RFS and OS for each patient based on the established model, we can therefore formulate an individualized treatment plan and improve the overall prognosis of patients.

At present, there is no standard treatment strategy for cervical neuroendocrine cancer. Considering the similarity of its histological morphology and biological behavior with those of small cell lung cancer, the clinical treatment for small cell lung cancer is often referenced [13,20,21]. In 2011, the Society of Gynecological Oncology (SGO) and the Gynecological Cancer Inter Group (GICG) had published expert consensus [22,23], suggesting that patients with cervical neuroendocrine cancer can adopt multimodal treatment including the combination of surgery, chemotherapy and radiotherapy. Radical resection of cancer lesions combined with adjuvant chemotherapy or radiotherapy is recommended for patients in early stage, while systemic chemotherapy or radiotherapy is recommended for patients with locally advanced and intolerability of surgery [21,24]. Among them, EP strategy (cisplatin + etoposide) is the first choice for small cell lung cancer at present, so it is widely used in patients with cervical neuroendocrine cancer, which can significantly improve the 3-year overall survival and progression-free survival rates of patients [6,25,26]. Moreover, studies also suggested that adjuvant radiotherapy for pelvic local lesion in patients at the early stage could improve local control and reduce the rate of distant metastasis [21,27]. In clinical practice, clinicians assess whether the patients are complicated with high risk factors correlated to poor prognosis to take appropriate postoperative adjuvant treatment programs. The nomogram model established in this study can objectively quantify the risk stratification of patients with cervical neuroendocrine carcinoma and provide a reliable theoretical basis for individualized evaluation of postoperative prognosis to generate individualized and comprehensive postoperative management strategies according to patients’ health status and recurrence risk. Previous studies have shown that the recurrence of cervical neuroendocrine carcinoma often occurs within 3 years after surgery. Therefore, this study determined the optimal cutoff value of 3-year RFS (85%) using the ROC curve, with patients classified under the high-RFS (RFS ≥ 85%) and low-RFS (RFS < 85%) groups. The results showed that the 3-year recurrence-free survival rate of patients under the high-RFS group (96.6%) was significantly higher than those from the low-RFS group (54.3%). This indicated that we should take active postoperative management for patients in the low-RFS group, including but not limited to actively taking adjuvant chemotherapy and radiotherapy or increasing the cycle of radiotherapy and chemotherapy; appropriately combining targeted therapy or immunotherapy; and making a stricter follow-up plan, instead of merely taking simple follow-up monitoring. For patients with high RFS, we can appropriately reduce the cycle of radiotherapy and chemotherapy and take a relatively easy follow-up program to reduce unnecessary waste of resources and improve the patients’ quality of life.

Although this study showed that pathological types, nerve invasion and endometrial involvement had no significant correlations with NECC recurrence, the results do not discount the importance of these clinical parameters in predicting the postoperative recurrence of cervical neuroendocrine carcinoma [15,19]. In fact, in other research studies, these factors have been verified as important indicators for predicting the postoperative recurrence of cancer, signifying that these factors were only potentially less significant as compared to the other high-risk factors we used in this study. In the past, it was suggested that the postoperative management should be decided according to whether the patients were complicated with some high-risk traditional clinical parameters, such as advanced stage, aggressive pathological types, lymph node metastasis and so on. This study confirmed that neuroendocrine markers had important value in predicting the postoperative prognosis of patients. Combining standard clinical factors with neuroendocrine markers was effective in improving the accuracy of prognostic prediction, significantly enabling the formulation of more effective treatment strategies. Therefore, clinicians should pay attention to NECC patients with positive immunohistochemistry, and active postoperative adjuvant therapy might be necessary even if these patients do not manifest invasive clinical parameters.

We have noted some limitations in this study. Firstly, in view of the rarity of cervical neuroendocrine carcinoma, the sample size of this study might be relatively small, which may partly cause statistical bias. A more conclusive validation could be achieved using a larger multicenter study with a more diverse set of patient characteristics to confirm the generalizability of our established model. Secondly, some other molecular markers (TP53, RB1) have also been reported and confirmed to be related to cancer recurrence, so it may be necessary to expand the screening of target proteins and explore new immunohistochemical or molecular markers to further improve the predictive ability of the model in future studies [3,8,28].

In conclusion, we have established a sufficiently accurate nomogram model that can predict the recurrence-free survival of cervical neuroendocrine carcinoma and provide a reliable reference tool for these patients to enable a more effective postoperative management.

## Figures and Tables

**Table 1:** The clinicopathological characteristics of the training group and validation group.

Variable	Training Cohort	%	Validation Cohort	%	*p* Value
	N = 171		N = 86		
Age median (years);					0.399
Mean ± SD	45.92 ± 9.991		44.91 ± 10.104		
Median (range)	46 (25–75)		45 (25–75)		
BMI (kg/m^2^);					0.781
Mean ± SD	22.81 ± 2.976		22.73 ± 2.605		
Median (range)	22.30 (18–39)		22 (18–32)		
FIGO stage					0.582
I	111	64.9	51	59.3	
II	24	14.0	16	18.6	
III	36	21.1	19	22.1	
Pathological type					0.527
LG-NECC	21	12.3	13	15.1	
HG-NECC	150	87.7	73	84.9	
Stromal invasion					0.567
<1/2	86	50.3	40	46.5	
≥1/2	85	49.7	46	53.5	
Endometrial invasion					0.912
Yes	23	13.5	12	14.0	
No	148	86.5	74	86.0	
Nerve invasion					
Yes	14	8.2	8	9.3	0.763
No	157	91.8	78	90.7	
C-UJI					0.168
Yes	40	23.4	27	31.4	
No	131	76.6	59	68.6	
LVI					0.681
Yes	45	26.3	22	25.6	
No	126	73.7	64	74.4	
LVSI					0.135
Yes	123	71.9	54	62.8	
No	48	28.1	32	37.2	
HPV					
negative	81	47.4	37	43.0	0.509
positive	90	52.6	49	57.0	
P16					0.681
0	21	12.3	13	15.1	
1+	80	46.8	44	51.2	
2+	12	7.0	4	4.7	
3+	58	33.9	25	29.1	
Syn					0.293
0	17	9.9	14	16.3	
1+	94	55.0	48	55.8	
2+	41	24.0	19	22.1	
3+	19	11.1	5	5.8	
CgA					0.523
0	53	31.0	20	23.3	
1+	72	42.1	42	48.8	
2+	21	12.3	9	10.5	
3+	25	14.6	15	17.4	
CD56					0.722
0	46	26.9	20	23.3	
1+	71	41.5	42	48.8	
2+	14	8.2	7	8.1	
3+	40	23.4	17	19.8	
Recurrence					0.122
Yes	23	13.5	18	20.9	
No	148	86.5	68	79.1	
Death					0.669
Yes	21	12.3	9	10.5	
No	150	87.7	77	89.5	
RFS (months)					0.815
Median	42		37.5		
Mean ± SD	52.73 ± 42.892		54.51 ± 43.767		
Range	2–150		4–145		
Follow-up (months)					0.803
Median	44		38		
Mean ± SD	54.19 ± 41.232		54.51 ± 43.763		
Range	2–150		4–145		

Abbreviations: BMI: body mass index; LG-NECC: Low grade-neuroendocrinal carcinoma of cervix; HG-NECC: High-grade neuroendocrinal carcinoma of cervix; LVSI: lymph vascular space invasion; LVI: lymph node involvement; CT: chemotherapy; RT: radiotherapy; FIGO: International Federation of Gynecology and Obstetrics; C-UJI: cervical–uterine junction invasion; SD: standard deviation.

**Table 2:** The univariate and multivariate COX regression analysis used to screen the risk factors of recurrence of cervical neuroendocrine carcinoma in training cohort.

Variable	Univariate Analysis			Multivariate Analysis		
	HR	95% CI	*p* Value	HR	95% CI	*p* Value
FIGO stage						
I	1.000		*p* < 0.001	1.000		0.023
II	3.903	1.100–13.847	0.035	8.868	1.523–15.652	0.015
III	8.718	3.305–15.996	*p* < 0.001	5.628	1.126–12.128	0.035
Stromal invasion (<1/2 vs. ≥1/2)	3.715	1.132–3.176	0.009	9.898	2.309–42.429	0.002
Nerve invasion (Yes vs. No)	3.367	1.131–10.024	0.029	1.185	0.144–9.776	0.875
LVSI (Yes vs. No)	4.857	1.138–20.727	0.033	7.077	1.099–5.564	0.039
LVI (Yes vs. No)	3.848	1.693–8.748	0.001	6.235	1.360–8.576	0.018
C-UJI (Yes vs. No)	3.466	1.513–7.938	0.003	8.693	2.606–15.445	0.005
CgA						
0	1.000		0.003	1.000		0.021
1+	6.300	0.788–5.380	0.083	6.302	1.143–6.841	0.040
2+	8.442	1.570–15.095	0.018	7.772	1.149–7.462	0.040
3+	9.673	2.933–17.216	0.003	9.362	4.304–10.180	0.003

Abbreviations: LVSI: lymph vascular space invasion; LVI: lymph node involvement; FIGO: International Federation of Gynecology and Obstetrics; C-UJI: cervical–uterine junction invasion.

**Table 3:** The discriminatory power (C-index) of different parameters models in the training cohort and validation cohort.

Variable	Training Cohort		Validation Cohort	
	C-Index	95% CI	C-Index	95% CI
FIGO stage, stromal invasion, lymph vascular space invasion, lymph node involvement, cervical uterine junction invasion	0.829	0.747–0.911	0.883	0.756–1.010
FIGO stage, stromal invasion, lymph vascular space invasion, lymph node involvement, cervical uterine junction invasion, CgA	0.863	0.784–0.942	0.884	0.758–1.010

## Data Availability

The data can be obtained upon request from the corresponding author and are not publicly available due to privacy or ethical restrictions.
